# Semantic Segmentation Method for Sparse Point Clouds Based on Straight Flow Completion and Multi-Feature Fusion

**DOI:** 10.3390/s26103056

**Published:** 2026-05-12

**Authors:** Tong Zheng, Zhiyuan Meng, Chongchong Yu, Tao Xie, Yewang Xu

**Affiliations:** School of Computer and Artificial Intelligence, Beijing Technology and Business University, Beijing 100048, China; zhengtong@buaa.edu.cn (T.Z.); zy15010727648@163.com (Z.M.); chongzhy@vip.sina.com (C.Y.); 15354203826@163.com (Y.X.)

**Keywords:** point cloud, semantic segmentation, completion, multi-feature fusion

## Abstract

Point cloud semantic segmentation is a vital task in 3D computer vision. However, the inherent sparsity of point clouds complicates the segmentation process. In contexts such as autonomous driving, moving objects frequently exhibit motion blur, which adversely affects semantic segmentation performance. These challenges hinder the practical application of point cloud semantic segmentation. To address these issues, this paper presents a novel semantic segmentation method that integrates sparse point cloud completion with multi-feature fusion. Specifically, the study emphasizes the development of efficient strategies for constructing and training point cloud completion models, aiming to expedite model parameter training while maximizing completion accuracy. Additionally, a semantic segmentation model is introduced that combines motion feature-enhanced instance features with semantic features, thereby enhancing adaptability to moving objects. Moreover, point cloud completion and semantic segmentation are linked in an end-to-end pipeline, facilitating accurate semantic segmentation of sparse point clouds in dynamic environments. During the experimental phase, publicly available Lidar point cloud datasets, including SemanticKITTI and the millimeter-wave radar dataset RADIal, are utilized to evaluate the proposed method against classical approaches in terms of point cloud completion performance and semantic segmentation effectiveness, thereby demonstrating the reliability of the proposed method.

## 1. Introduction

Point cloud semantic segmentation is a critical task in 3D computer vision that involves assigning a semantic label to each point within a point cloud [1,2]. As fields such as autonomous driving, robotics, and AR/VR rapidly advance, point cloud semantic segmentation has emerged as a vital technology. The depth and positional information inherent in 3D point clouds render them more effective for scene understanding compared to 2D image semantic segmentation. However, the inherent sparsity of point clouds results in significantly increased computational demands and resource consumption when directly applying 2D convolution techniques to 3D data. As a result, much of the research has concentrated on overcoming the challenges associated with point cloud data [3]. Currently, approaches to point cloud semantic segmentation can be divided into 3D and 4D point cloud semantic segmentation. Within this framework, 3D point cloud semantic segmentation can be further categorized into projection-based methods, voxel-based methods, point-based methods, and hybrid methods, based on the representation form of the point cloud.

Projection-based methods entail the projection of 3D point clouds onto 2D planes, followed by the application of deep learning networks to extract features, which are subsequently back-projected into the 3D point cloud to yield segmentation results. These methods demonstrate robust scalability and satisfy real-time requirements. Current research in this domain is primarily categorized into two types: multi-view projection and spherical projection. A notable example of multi-view projection is Multi-view Convolutional Neural Networks (MVCNN) [4]. This approach inputs multiple 2D images into a 2D convolutional network and utilizes max pooling to aggregate features from the projected images, thereby facilitating 3D object segmentation tasks. However, the efficacy of multi-view projection is highly contingent upon the selection of viewpoints, and both structural and geometric information may be compromised during the projection process. To mitigate these challenges, SqueezeSeg [5] employs spherical projection to convert 3D point clouds into images. It then utilizes the lightweight convolutional neural network SqueezeNet for feature extraction. Ultimately, the segmentation results are projected back into the 3D point cloud. Building on this foundation, the SqueezeSegV2 network [6] incorporates a context aggregation module, which enhances the network’s ability to capture contextual information through a larger receptive field. Although spherical projection methods can capture more structural and geometric information while simplifying the overall inference framework, they are unable to process point clouds directly, thereby hindering the full exploitation and exploration of the geometric and topological information inherent in 3D point clouds.

Voxel-based methods convert 3D point clouds into voxel representations prior to processing with 3D convolutional operations. However, the inherent sparsity of point cloud data results in numerous empty voxels during this conversion. When these empty voxels are processed through dense 3D convolutional networks, they create significant computational overhead, which constrains the advancement of such networks. As a result, many studies have concentrated on utilizing sparse convolution techniques to mitigate this issue. Graham et al. [7] introduce a submanifold sparse convolution method that alleviates the computational burden of empty voxels by omitting them during the convolution process. Cylinder3D [8], which considers the properties of LiDAR point cloud data, divides the 3D space into cylindrical partitions and performs voxelization to achieve a more balanced point distribution. Another sparse convolution strategy involves the construction of specialized discrete spaces using tree-based structures. For example, OctNet [9] utilizes a set of unbalanced octrees to index the point cloud, thereby expediting 3D convolution operations on point clouds.

Point-based methods represent the most straightforward approach to point cloud processing. Building on PointNet [10], these methods emphasize the extraction of robust spatial features. PointNet++ [11] utilizes farthest point sampling for downsampling and organizes points through a K-Nearest Neighbors (KNN) mechanism. To mitigate the disorder of point sets, PointCNN [12] executes convolution based on the relative positions and distances of points, thereby capturing local spatial information. Kernel Point Convolution (KPConv) [13] introduces a more adaptable kernel point convolution mechanism, defining a spherical region around kernel points and applying deformable 3D convolution kernels to aggregate features from neighboring points. While these methods effectively extract local features through convolutional operations, they also learn the local structures of point clouds, enhancing overall network performance. Nonetheless, the significant computational cost associated with these approaches restricts their applicability in large-scale outdoor scenarios.

Integrating multiple semantic segmentation methods can capitalize on their individual strengths, thereby improving the overall performance of the final model. For example, Grid-GCN [14], a graph convolutional network (GCN)-based approach, utilizes a volumetric technique known as coverage-aware grid query, which achieves enhanced performance while simultaneously reducing computational costs. The CrossModalSync method, which facilitates joint temporal-spatial fusion for semantic scene segmentation in large-scale environments, incorporates projection and multi-scale convolution, temporal alignment, and a cross-modal fusion network featuring residual modules. By merging LiDAR and camera data, this approach significantly improves segmentation performance in dynamic and complex settings [15]. Furthermore, the Multilateral Cascading Network (MCNet) is specifically tailored for large-scale outdoor point clouds, effectively addressing challenges related to point cloud sparsity, high redundancy, and class imbalance [16].

Based on existing research, this paper examines methods to enhance the semantic segmentation of sparse point clouds associated with moving targets. The primary contributions are as follows:

(1) To tackle the challenges posed by sparse point cloud data, this study investigates Completion techniques that improve resolution while optimizing parameter learning efficiency.

(2) To address the difficulties in extracting and representing motion features, we explore the integration of motion feature extraction with traditional point cloud semantic segmentation, thereby improving segmentation performance for moving objects.

(3) The proposed methods are assessed using the SemanticKITTI LiDAR and RADIal radar point cloud datasets, with experimental analyses validating their efficacy and reliability.

The structure of the paper is organized as follows: Section 2 reviews related works on point cloud completion and motion feature extraction. Section 3 outlines the strategy of the proposed method. Section 4 presents experiments designed to verify the effectiveness of the proposed method. Finally, Section 5 summarizes the research findings of this paper.

## 2. Related Works

Under this context, this paper focuses on reviewing relevant work in the areas of point cloud completion and motion feature extraction, which serves as the foundation for our subsequent research.

### 2.1. Point Cloud Completion Methods

The widespread adoption of 3D sensors, including LiDAR and Kinect, has made the acquisition of point cloud data increasingly accessible. Nonetheless, point clouds frequently suffer from incompleteness due to factors such as limited sensor resolution, occlusion, and scanning inaccuracies. Consequently, effectively completing these partial point clouds and enhancing their representational capacity has become a significant research challenge [16]. Early methods for point cloud completion predominantly relied on geometric models. These approaches typically employed surface reconstruction algorithms, including Poisson Surface Reconstruction (PSR) and Delaunay triangulation [17]. Such algorithms generate smooth surfaces to fill in missing regions by interpolating or fitting the geometric structures present within the point cloud. However, these methods are sensitive to noise, struggle to accurately capture complex geometries, heavily rely on pre-existing model databases, and demonstrate limited performance and scalability when processing large-scale sparse point clouds.

The emergence of deep learning has encouraged researchers to apply it to point cloud completion tasks, resulting in the creation of numerous neural network-based methods. By capturing the geometric characteristics of point cloud data, these approaches yield more detailed and realistic completion outcomes. Completion methods can be broadly classified into several categories: point-based, convolution-based, graph-based, folding-based, Transformer-based methods, and generative model-based approaches. The latter category can be further subdivided into methods utilizing Variational Autoencoders (VAEs), Generative Adversarial Networks (GANs), and diffusion models.

Point-based methods represent a classical approach to point cloud processing, yet they often entail significant computational costs and require substantial computing power. Notably, PointNet [10] and PointNet++ [11] are among the most prominent techniques for extracting point-wise features. PointNet++ specifically enhances the ability to capture local geometric structures by hierarchically learning point cloud features through local sampling and aggregation of point sets. Furthermore, the PF-NET developed by Huang et al. [18] generates high-quality point clouds by preserving the spatial arrangement of the input partial point cloud and predicting the geometric structure of missing regions. This method also incorporates a discriminator to evaluate point cloud quality, utilizing adversarial loss to enhance the realism of the output. Additionally, SFA-Net [19] addresses detail loss through multi-scale feature extraction, aggregating global, local, and residual features, followed by coordinate-based uniform distribution reconstruction, which aids in reducing noise and outliers.

Inspired by the success of convolutional neural networks (CNNs) in two-dimensional image processing, several methods have been adapted for point cloud completion. For example, Hua et al. [20] designed convolution kernels on regular three-dimensional grids, assigning identical weights to points within the same grid cell. Thomas et al. [13] developed a KPConv module that utilizes learnable kernels to process three-dimensional point clouds, accommodating both rigid and deformable configurations. With the advent of point cloud voxelization, subsequent studies have demonstrated that voxelized representations are more effective for shape completion using CNNs. Wang et al. [21] introduced a Voxel-Edge Generation Network (VE-PCN) that processes voxel structures to generate fine-grained details. However, CNN-based completion methods that rely on voxelized or grid-based representations necessitate converting point clouds into alternative data structures. This conversion often introduces sensitivity to point cloud resolution and may distort spatial relationships between points, ultimately resulting in suboptimal completion performance.

Both graphs and point clouds are classified as non-Euclidean structural data. By conceptualizing points or local regions as nodes within a graph, graph-based processing techniques can be effectively applied to point clouds. Among these methodologies, DGCNN [22] stands out as a pioneering study that utilizes dynamic graph convolution to extract relationships among nodes and compute adjacency matrices. Furthermore, Shen et al. [23] introduce a graph-guided deformation network, which considers the input data as control points and the intermediate generated results as support points. This approach simulates a least-squares Laplacian deformation process via mesh deformation, allowing learned mesh details to be updated in accordance with the input graph and adjusted adaptively. Nevertheless, existing networks continue to face challenges in fully exploiting the spatial and geometric relationships between each point and its neighbors, leading to suboptimal performance in completion tasks.

Folding-based point cloud completion methods create complete point cloud shapes by mapping 2D grids into 3D space. Yang et al. [24] were the first to propose and demonstrate that this approach could function as a general architecture for point cloud completion. Xie et al. [25] introduced SpareNet, which utilizes a style-based adversarial rendering point generator for completion. This framework incorporates channel-wise attention-based edge convolution during point feature extraction, effectively integrating local and global features while enhancing point generation through style-based folding. However, these methods typically depend on a single global shape to predict the entire point cloud, thereby neglecting rich local geometric information. This limitation can result in issues such as uneven point distribution, blurred details, and structural loss, ultimately compromising the quality of completion.

The point Transformer [26] is the first method to effectively adapt the Transformer architecture for processing point clouds. Leveraging the robust representational learning capabilities of the Transformer, Liu et al. [27] developed a multi-scale point cloud completion framework that incorporates Transformer modules. Their approach combines attention mechanisms with feature embedding layers to extract point cloud features at various resolutions. Furthermore, an attention-based discriminator is introduced in the decoder to enhance the network’s completion performance. Nevertheless, the substantial number of parameters associated with Transformer models presents challenges for practical applications.

Early efforts in generative model-based point cloud completion predominantly employed Variational Autoencoder (VAE) or Generative Adversarial Network (GAN) frameworks. For example, Spurek et al. [28] present a variational autoencoder architecture called Hyper Pocket, which is constructed using the hypernetwork paradigm. Wang et al. [29] introduce a GAN-based generator that implements a cascaded refinement strategy, enabling the synthesis of high-density point clouds through a coarse-to-fine approach. In 2020, the Denoising Diffusion Probabilistic Model (DDPM) [30] was proposed, which also found applications in point cloud completion tasks. Notably, Luo et al. [31] developed a probabilistic model for point cloud generation by conceptualizing points as particles and training the model through forward and reverse diffusion processes that connect the initial distribution to a noise distribution. The reverse process, which transitions from noise to the target distribution, is identified as the procedure for generating point clouds. Another contemporaneous work, PVD [32], introduces a diffusion-based approach for point cloud generation that utilizes a hybrid point-voxel representation to process shapes. This method enables unconditional generation without the need for an additional shape encoder. Other notable methods in this category include SoftFlow [33] and PointFlow [34]. Although generative models exhibit strong performance in point cloud synthesis, they often entail high training costs, demand substantial computational resources, and present lengthy inference times, which complicates their application in real-time scenarios. In response to these challenges, this paper explores parameter learning strategies within the generative model-based point cloud completion framework, with the objective of improving completion accuracy while expediting model learning.

### 2.2. Point Cloud Motion Feature Extraction Method

In practical applications, single-frame point cloud semantic segmentation methods are often limited by the lack of motion information, which significantly hinders continuous online processing of objects with the same semantic labels. In the context of autonomous driving, encounters between vehicles traveling in opposite directions or during overtaking maneuvers can result in significant motion blur and smearing caused by high-speed oncoming cars. This phenomenon leads conventional segmentation models to erroneously classify the “car” as “background” [35]. Such misclassifications represent a critical issue that warrants attention. To address this issue, the emerging field of 4D semantic perception has attracted increasing attention, as it enables the extraction of motion features from point clouds.

Typical 4D CNNs or Recurrent Neural Networks (RNNs), such as MinkowskiNet [36], generalize 2D convolution functions into 4D sparse convolutions and correspondingly organize 4D point cloud data into sparse tensors to facilitate the construction of 4D deep neural networks, thereby achieving 4D spatiotemporal perception. However, its scalability is limited. As the number of points and data frames increases, computational costs grow rapidly. Furthermore, P4Transformer [37] incorporates point-based 4D convolution and transformers to fuse spatial and temporal information. Nevertheless, this network requires heavy downsampling of point clouds, making it difficult to apply to real-time outdoor point cloud processing. Additionally, SpSequenceNet [38] inputs two consecutive frames of point cloud data into a sparse 3D convolutional neural network for spatial feature extraction, employing cross-frame global attention and cross-frame local interpolation modules to extract temporal correlation features. However, this method overlooks the issue of spatial coordinate inconsistency caused by ego-motion during data acquisition, resulting in limited temporal feature extraction capability. Duerr et al. [39] propose a recurrent structure that optimizes segmentation performance through spatial and temporal alignment. But this method incurs high computational costs and exhibits low utilization of temporal information. SVQNet [40] refines unseen geometric information in the current frame by searching historical local features around each point. Moreover, 2DPASS [41] is built upon distillation learning, transferring image knowledge to assist point cloud segmentation. TASeg [42] selects specific time steps for stacking multiple scans based on the classification difficulty of each category, while also leveraging temporal image features to enhance point features. SegNet4D [43] decomposes the multi-frame point cloud semantic segmentation task into single-frame point cloud semantic segmentation and moving object segmentation, improving multi-frame segmentation accuracy by fusing per-point semantic predictions and motion states. The aforementioned methods are all developed for relatively dense LiDAR point clouds and have achieved notable performance. However, once the point cloud resolution decreases, particularly in cases similar to 4D millimeter-wave radar point clouds, these methods struggle to achieve high-precision semantic segmentation results due to insufficient spatiotemporal and instance information.

## 3. Methodology

**Figure 1:**
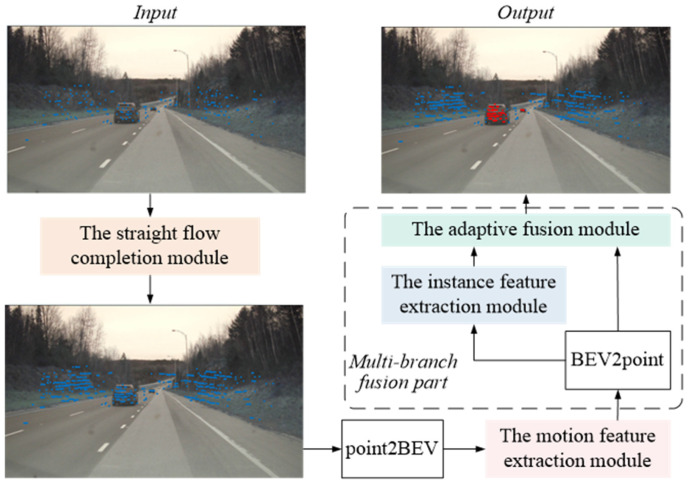
The overall structure of the proposed model.

The overall architecture of the sparse point cloud semantic segmentation method proposed in this paper is illustrated in Figure 1. Initially, a straight flow-based point cloud completion process is applied to the sparse point cloud data to obtain more comprehensive point cloud information. Furthermore, since the proposed method primarily targets the object semantic segmentation task in autonomous driving scenarios, the motion characteristics of objects are also non-negligible. Therefore, the model extracts motion feature information from the completed point cloud data and enhances the point cloud representation. Subsequently, after processing by a multi-branch feature extraction module, both semantic and instance features of the point cloud are acquired. The final semantic segmentation results are obtained through the processing of an adaptive fusion module. The subsequent sections will provide detailed introductions to each module.

### 3.1. The Learning Process of Straight Flow Completion Module

**Figure 2:**
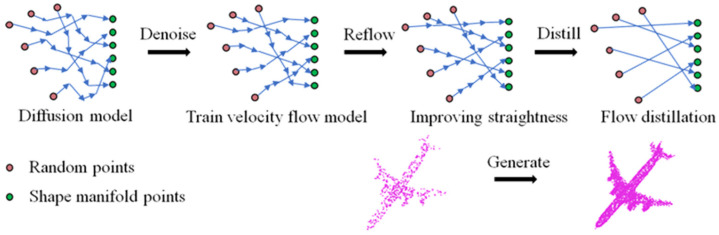
The learning process of the straight flow-based point cloud completion module.

The learning process of the straight flow-based point cloud completion module proposed in this paper is illustrated in Figure 2. Through this module, the 3D point cloud completion can be achieved in a single step. The point cloud completion process can be regarded as transforming the incomplete point cloud into the target point cloud data by learning trajectories. First, we construct an ordinary differential equation representing the shortest transmission path, specifically a neural velocity flow network, and employ a neural network for point cloud denoising. Next, we implement a reflow process to straighten the learned trajectories, followed by a distillation process to enhance the step size of the learning procedure, facilitating rapid model updates and iterations. Finally, we extract the objective of the neural network to ensure that a single update with a fixed large step size achieves the same effect as multiple iterations of smaller updates.

Let $X_{0}\in R^{M\times 3}$ be a point cloud dataset that follows a Gaussian distribution and has spatial coordinates (x, y, z) as its independent variables. Moreover, $X_{1}\in R^{M\times 3}$ is the real data. Define $\nu_{\theta }$ as a velocity field network that adheres to the following ordinary differential equation.

(1) $$\underset{drift}{\underset{︸}{dX_{t}}}=\underset{velocity}{\underset{︸}{\nu_{\theta }\left(X_{t},t\right)}}\underset{\mathrm{time}\;\mathrm{interval}}{\underset{︸}{dt}},t\in \left[0,1\right]$$

where $X_{t}$ is the intermediate point cloud state of time t. $\nu_{\theta }$ is a neural network with parameter θ. $\nu_{\theta }$ enables $X_{t}$ to transform into $X_{1}$. At any given moment, the optimal direction for learning is from $X_{1}$ to $X_{0}$. Therefore, the speed field can be made to conform to the optimal ordinary differential equation through the following optimization processing, i.e., $dX_{t}=\left(X_{1}-X_{0}\right)dt$.

(2) $$\underset{\theta }{arg\min}\int_{0}^{1}E\left[{\left\|\nu_{\theta }\left(X_{t},t\right)-\left(X_{1}-X_{0}\right)\right\|}^{2}\right]dt$$

where $X_{t}=tX_{1}+\left(1-t\right)X_{0},t\in \left[0,1\right]$. For each data sample $X_{1}$, it is necessary to find $X_{0}$ from the Gaussian noise, find the time *t* from [0, 1], and minimize the following equivalent loss.

(3) $$\underset{\theta }{arg\min}E\left[{\left\|\nu_{\theta }\left(X_{t},t\right)-\left(X_{1}-X_{0}\right)\right\|}^{2}\right],t∼U\left(0,1\right)$$

After the neural velocity field is trained, new samples can be generated by discretizing the ordinary differential equation using the Euler solver in Equation (1), namely

(4) $$X{’}_{\left(\hat{t}+1\right)/N}\leftarrow X{’}_{\hat{t}/N}+\frac{1}{N}\nu_{\theta }\left(X{’}_{\hat{t}/N},\frac{\hat{t}}{N}\right)$$

where time interval is defined as $\hat{t}\in \left\{0,1,\ldots ,N-1\right\}$. $X{’}_{0}=X_{0}$ is the generated samples. It can be clearly seen that the larger the *N* value is, the higher the accuracy of the solver will be.

In the aforementioned steps, network $\nu_{\theta }$ is trained based on $\left(X_{0},X_{1}\right)$. Here, $X_{1}$ represents the ground truth. After verification, it was found that the learning trajectory in this way is still curved. Therefore, in order to accurately approximate Equation (1), a very large *N* is required in Equation (4). To reduce the value of *N*, even if the learning process is approximated as a linear flow, the Gaussian noise can be sampled under a fixed network, generating samples $X{’}_{0}$ that follow Equation (4), and then the $\left(X{’}_{0},X{’}_{1}\right)$ data can be used to replace $\left(X_{0},X_{1}\right)$ in Equation (2). This is the reflow processing procedure mentioned in Figure 1. In this case, the transmission cost is defined as the form, and the straightness of the trajectory can be expressed as

(5) $$Straightness=\frac{1}{N}\sum_{\hat{t}=0}^{N-1}\left[{\left\|\left(X{’}_{1}-X{’}_{0}\right)-\nu_{\theta }\left(X_{\hat{t}/N},\hat{t}/N\right)\right\|}^{2}\right]$$

The process of straight flow learning is such that at any given moment, the velocity value $\nu_{\theta }\left(X_{\hat{t}/N},\hat{t}/N\right)=X{’}_{1}-X{’}_{0}$ is fixed at $\hat{t}$. At this point, the straightness of the trajectory is zero.

(6) $$X{’}_{1}=X{’}_{0}+\nu_{\theta }\left(X{’}_{0},0\right)$$

Obviously, our goal is to ensure that the quality of the updated samples in Equation (6) is similar to the samples generated by Equation (4) when *N* is large. Therefore, the value of *N* needs to be reduced through the distillation processing. The objective of the distillation processing is designed as:

(7) $$\underset{\theta }{\arg\min}E\left[\mathrm{Dist}\left(\underset{onestep}{\underset{︸}{X{’}_{0}+\nu_{\theta }\left(X{’}_{0},0\right)}},\underset{Nstep}{\underset{︸}{X{’}_{1}}}\right)\right]$$

where $X{’}_{1}$ represents the point cloud generated during the backflow process. $\mathrm{Dist}\left(\cdot \right)$ is the loss function used to measure the difference between two point-clouds, and it can be expressed using the Chamfer Distance (CD), that is

(8) $$CD\left(X_{i},X_{j}\right)=\sum_{p\in X_{i}}\underset{\hat{p}\in X_{j}}{\arg\min}{\left\|p-\hat{p}\right\|}_{2}+\sum_{\hat{p}\in X_{j}}\underset{p\in X_{i}}{\arg\min}{\left\|p-\hat{p}\right\|}_{2}$$

where $p\in X_{i}$ and $q\in X_{j}$ are the point clouds from two point-clouds clusters.

### 3.2. The Structure of Motion Feature Extraction Module

The motion feature extraction module in this paper can be divided into two components: a temporal dynamic feature extraction branch and a semantic feature extraction branch. The outputs of these two branches are then concatenated and processed through a channel attention mechanism to ultimately yield the motion features. Prior to extracting motion features, it is necessary to transform the multi-frame consecutive point clouds into the viewpoint space for alignment. Subsequently, the aligned point clouds are projected into a Bird’s-Eye View (BEV) image, and the residual between past and current BEV images is computed to extract traces left by moving objects.

Specifically, suppose the point cloud of the current frame consists of *M* points, which is represented as $S_{0}={\left\{p_{i}\in R^{4}\right\}}_{i=1}^{M}$, whose homogeneous coordinates is $p_{i}={\left[x_{i},y_{i},z_{i},1\right]}^{T}$. Additionally, the mapping relationship between *N* consecutive point clouds $S_{0},S_{1},S_{2},\ldots ,S_{N-1}$ is defined as $T_{1}^{0},T_{2}^{1},T_{3}^{2},\ldots ,T_{N}^{N-1}$. The past *N* − 1 consecutive point clouds are transformed into:

(9) $$S_{j\to 0}=\left\{\left.p{’}_{i}=T_{j}^{0}p_{i}\right|p_{i}\in S_{j}\right\},T_{j}^{0}=\prod_{k=0}^{j-1}T_{j-k}^{j-1-k}$$

Project the aligned point clouds into a single-channel BEV image. Limit the coordinate values of each point as follows: $x{’}_{j}\in \left[X_{\min},X_{\max}\right]$, $y{’}_{j}\in \left[Y_{\min},Y_{\max}\right]$ and $z{’}_{j}\in \left[Z_{\min},Z_{\max}\right]$. Then it is transformed into the column space.

(10) $$\left\{\begin{array}{c} I_{\left(u,v\right),j}=\left\{\left.z{’}_{j}\right|z{’}_{j}\in p{’}_{j}\right\}\\ u=\left[\frac{x{’}_{j}-X_{\min}}{g}\right]\\ v=\left[\frac{y{’}_{j}-Y_{\min}}{g}\right] \end{array}\right.$$

where $I_{\left(u,v\right),j}$ represents the height at which the aligned point cloud is located relative to point $\left(u,v\right)$. *g* is the grid resolution. Subsequently, the points in the column space are mapped onto a single-channel BEV image with a size of *H* × *W*. Namely, each pixel is represented as $B_{\left(u,v\right),j}$. Its value is the difference between the maximum and minimum heights of each pillar, that is

(11) $$B_{\left(u,v\right),j}=\max\left[I_{\left(u,v\right),j}\right]-\min\left[I_{\left(u,v\right),j}\right]$$

Calculate the residuals for the BEV images of the previous *N* − 1 frames and the current frame respectively

(12) $$R_{\left(u,v\right),j\to 0}=B_{\left(u,v\right),0}-B_{\left(u,v\right),j},j\in 1,2,\ldots ,N-1$$

**Figure 3:**
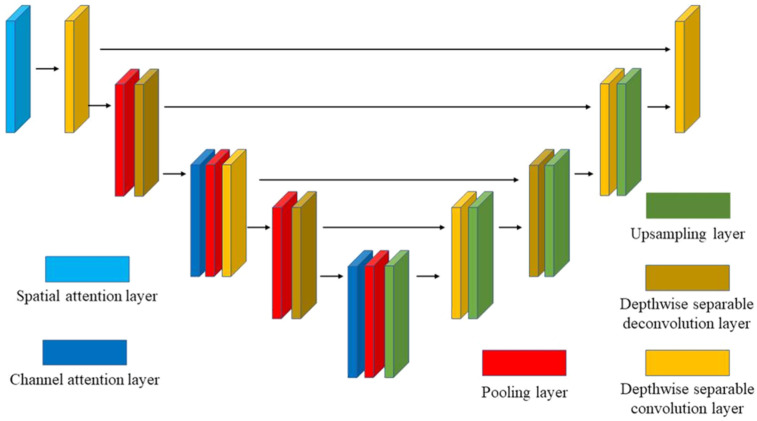
The structure of semantic feature extraction branch.

The semantic feature extraction branch is structurally divided into an encoder and a decoder, as illustrated in Figure 3. The encoder consists of multiple convolutional layers and attention modules, progressively extracting high-level feature representations. To ensure high accuracy while minimizing the parameter count and computational complexity, this section employs Depthwise Separable Convolution (DSC) for deep feature extraction. Furthermore, to enhance the focus on salient features and improve the effectiveness of feature representation, this paper introduces a hierarchical attention mechanism. This mechanism combines channel attention and spatial attention to adjust weights across different feature dimensions, enabling the model to extract critical information from complex millimeter-wave radar point cloud data. During the decoder process, the spatial resolution of feature maps is gradually restored through upsampling and convolutional operations, generating fine-grained segmentation results. At each upsampling stage of the decoder, the corresponding encoder feature maps are concatenated with the decoder feature maps along the channel dimension, followed by convolutional layers for feature fusion, thereby implementing skip connections. This approach allows the decoder to leverage low-level detail information from the encoder, preventing the loss of important spatial details during downsampling.

### 3.3. The Structure of Multi-Branch Fusion Part

The multi-branch fusion module transcends a mere concatenation of features from various channels; it integrates motion features with instance features. Initially, the motion features are converted from the bird’s-eye view (BEV) space to the point level, constituting the first fusion branch. Following this, the point-level motion features undergo clustering. Additionally, the average points for each instance category are extracted to form the output instance features. Ultimately, an adaptive fusion module is utilized to combine the point-level features from both branches, resulting in the final semantic segmentation outcomes.

**Figure 4:**
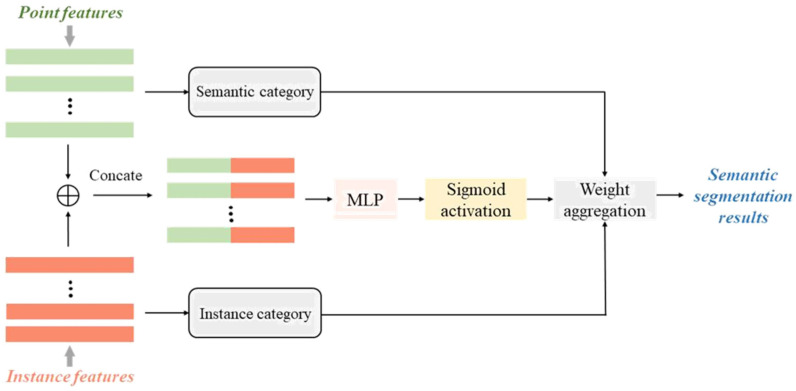
The structure of adaptive fusion module.

In the adaptive fusion module, the proposed model adaptively fuses the predictions from multiple branches based on the confidence scores of the two types of features, thereby obtaining spatio-temporally consistent segmentation results. The fusion architecture is illustrated in Figure 4. For features from different branches, a simple concatenation fusion is first performed. The point features are then connected along the channel dimension. The confidence scores are computed using two MLPs with non-shared weights and a sigmoid activation function, yielding values ranging from 0 to 1. Finally, the predictions from the two branches are weighted according to their confidence scores. The process of computing the confidence scores is as follows:

(13) $$S=sigmoid\left(MLP\left(conact\left(H_{seg},H_{ins}\right)\right)\right)$$

where $H_{seg}$ and $H_{ins}$ represent the point features and instance features extracted by the previous network, respectively. Then, the predicted features of the two branches are adaptively merged using the obtained confidence scores.

(14) $$P^{final}=\left(1-S\right)\cdot P_{seg}+S\cdot P_{ins}$$

where $P_{seg}$ and $P_{ins}$ are the further extracted features for semantic features $H_{seg}$ and instance features $H_{ins}$, respectively.

## 4. Experiments and Analysis

### 4.1. Experiments Settings and Dataset

(1) Dataset

To validate the semantic segmentation performance of the proposed model in this paper, we conducted experiments and analysis using a publicly available dataset, namely SemanticKITTI. This dataset, developed by a research team from the University of Bonn in 2019, is a large-scale outdoor scene dataset based on automotive LiDAR, built upon the renowned KITTI Vision Benchmark. The dataset comprises 22 sequences (with 100,000 points per scan), where sequences 00 to 10 are designated as the training set, and sequences 11 to 21 as the test set. It provides 23,201 complete 3D scans for training and 20,351 scans for testing. In accordance with the definitions in the semantic-kitti.yaml file, categories, such as “outlier”, “other-structure” and “other-object”, are grouped into the “unlabeled” class, while categories, including “bus”, “on-rails” and “moving-other”, are classified under the “other-vehicle” class.

**Figure 5:**
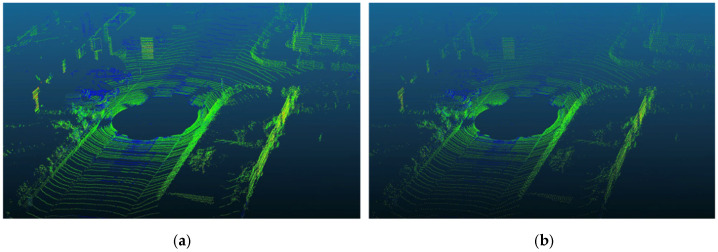
Comparison of point cloud imaging results before and after sparse processing in the same scene, (**a**) original cloud point, (**b**) sparse cloud point.

From the perspective of imaging results, the point clouds in these datasets are generally dense, making it difficult to evaluate the effectiveness of the proposed method on sparse point clouds. To address this, we design a sparsification pipeline based on the publicly available LiDAR point cloud dataset SemanticKITTI, establishing the Sparse-SemanticKITTI dataset to analyze the validity and reliability of the proposed method. Specifically, by leveraging the bin-format and label-format files contained in the sequence/velodyne folder of the original KITTI Odometry Benchmark, we manually perform sparsification operations in a C++ environment using the PCL library. The sparsified point cloud retains approximately one-third of the original data volume, while all other data categories remain unchanged. Interval sampling utilizing LiDAR scan lines is employed to replicate the sparse point cloud characteristics of distant targets. Visual comparisons are conducted to verify the consistency between the distribution of sparse data and real-world scenarios. By importing the Sparse-SemanticKITTI data under both scenarios into point cloud visualization software and comparing it with the corresponding SemanticKITTI data, the results are shown in Figure 5. The left side displays the original data from SemanticKITTI, with 124,668 points, while the right side shows the Sparse-SemanticKITTI data, where the point count is reduced to 41,556. As can be observed, objects such as vehicles, road surfaces, and trees exhibit noticeable sparsity. After batch processing, the entire SemanticKITTI dataset was subjected to the same treatment, resulting in the complete Sparse-SemanticKITTI dataset.

Furthermore, this paper utilizes the publicly available RADIal dataset to validate the performance of the point cloud completion model. The RADIal dataset comprises two hours of vehicle driving data, collected in urban streets, highways, and rural road environments. The sensors employed include cameras, LiDAR, and 4D millimeter-wave radar, along with the GPS position and driving information. The dataset includes 91 video sequences, which meticulously document the vehicle’s operation across various locations and environmental conditions. The 4D millimeter-wave radar in the dataset consists of 12 transmit antennas and 16 receive antennas, forming a 192-virtual-antenna array. This antenna configuration facilitates high-resolution measurements in both horizontal azimuth and vertical elevation angles.

(2) Assessment metric

To evaluate the point cloud completion and semantic segmentation performance, this paper adopts the following assessment metric. In the evaluation stage of point cloud completion, common similarity metrics include CD, Hausdorff Distance (HD), Earth Mover’s Distance (EMD), and Fréchet Point Cloud Distance (FPD), among others. Among these, the CD is a method used to measure the distance between two point clouds and has been widely applied in fields such as computer vision, computer graphics, robotics, and autonomous driving. The CD is computed by calculating the distance from each point in one point cloud to its nearest neighbor in the other point cloud, followed by averaging these distances to obtain the CD value. The definition is given by Equation (8).

Additionally, the HD describes the distance between subsets in a metric space, measuring the maximum degree of mismatch between two point sets by calculating the maximum distance from a point in one set to its nearest neighbor in the other set. For point cloud data, to enhance the effectiveness of the metric evaluation, the maximum distance is replaced with the average distance, the expression of which is as follows:

(15) $$\left\{\begin{array}{c} h\left(A,B\right)={\mathrm{mean}}_{a\in A}\min_{b\in B}\left\|a-b\right\|\\ h\left(B,A\right)={\mathrm{mean}}_{b\in B}\min_{a\in A}\left\|b-a\right\|\\ HD\left(A,B\right)=\max\left(h\left(A,B\right),h\left(B,A\right)\right) \end{array}\right.$$

where *A* and *B* respectively represent two sets of points. a∈A and b∈B indicates the points in two sets of points. Moreover, $h\left(A,B\right)$ and $h\left(B,A\right)$ represent one-way HD. $\left\|a-b\right\|$ and $\left\|b-a\right\|$ are the Euclidean distance between two points. $H\left(A,B\right)$ is bidirectional HD.

In the field of 3D point clouds, the EMD measures the similarity between different point clouds by computing the minimal distance between corresponding points through a mapping from the ground truth to the generated point cloud. The mathematical expression is as follows:

(16) $$EMD\left(A,B\right)=\min_{\phi :A->B}\frac{1}{\left|A\right|}\sum_{x\in A}\left\|x-\phi \left(x\right)\right\|$$

where ϕ:A→B is the mapping between two point sets. x∈A represents the points in the point set before point cloud generation.

In point cloud data evaluation, FPD measures the similarity between the ground-truth point cloud and the generated point cloud by computing the 2D distance in the feature space. The mathematical expression is as follows:

(17) $$FPD\left(A,B\right)={\left\|m_{A}-m_{B}\right\|}_{2}^{2}+T_{r}\left(\sum A+\sum B-2{\left(\sum A\sum B\right)}^{\frac{1}{2}}\right)$$

where $m_{A}$ and ∑A are the mean vector and covariance matrix calculated from the actual point cloud data. $m_{B}$ and ∑B are the mean vector and covariance matrix calculated from the generated point cloud data. $T_{r}\left(\cdot \right)$ represents the total sum array obtained by summing the elements along the main diagonal of the matrix.

F1-score is a crucial classification evaluation metric that provides a comprehensive assessment of model performance by simultaneously considering both the precision and recall of the classification model. Precision measures the probability that samples predicted as positive are actually positive. For the point cloud completion task, F1-score serves as a comprehensive metric that balances both precision and recall, thereby evaluating the matching degree between the completed point cloud and the ground truth. Evidently, a higher F1-score indicates superior completion performance of the model. The specific expression is as follows:

(18) Precisiond=1nB∑b∈Bmina∈Aa−b<dRecalld=1nA∑a∈Amina∈Aa−b<dF1d=2⋅Precisiond⋅RecalldPrecisiond+Recalld

where $\mathrm{Precision}\left(d\right)$ and Recalld are precision and recall when the distance threshold is d. $n_{A}$ and $n_{B}$ represent the number of points in two different sets of points.

In the evaluation stage of point cloud semantic segmentation, this study employs the Intersection-over-Union (IoU) metric for moving objects as the evaluation criterion. The expression is as follows:

(19) $$IoU=\frac{TP}{TP+FP+FN}$$

where TP, FP and FN are true positive, false positive, false negative, respectively. Furthermore, the mean IoU (mIoU) across all categories can also be employed as an evaluation metric.

(3) Hyperparameters and environment configuration

The experimental settings for model training are presented in Table 1. In addition, the experimental environment is detailed in Table 2. The model is trained for 120 epochs on a single GeForce RTX 2080 Ti GPU with a batch size of 5. The Adam optimizer is employed to minimize the overall loss in the loss function, with the learning rate initialized at 0.01 and decayed by a factor of 0.5 every 10 epochs.

### 4.2. The Effect of Point Cloud Completion

In this section, both classical point cloud completion methods and the proposed straight flow completion model are applied to the same Sparse-SemanticKITTI dataset for point cloud completion. A comparison of the completion results is presented in Table 3. The classical completion methods include SoftFlow [31], PointFlow [32], and PVD [30]. The evaluation metrics employed for point cloud completion consist of the aforementioned CD, HD, EMD, FPD, Precision, Recall and F1-score.

As can be observed from the results in Table 3, the straight flow completion module proposed in this paper achieves optimal point cloud completion performance across all seven evaluation metrics. Particularly on the FPD metric, the proposed method outperforms the best-performing classical method by a significant margin of approximately 4.54. The proposed approach also demonstrates superior performance in the three classification evaluation metrics, showing an improvement of 8.62 in Precision compared to the best classical method. Furthermore, it maintains a competitive advantage in the comprehensive F1-score metric, where the overall evaluation advantage is most pronounced. In conclusion, quantitative comparisons confirm that the proposed method exhibits distinct advantages over classical point cloud completion approaches in the point cloud completion task.

**Figure 6:**
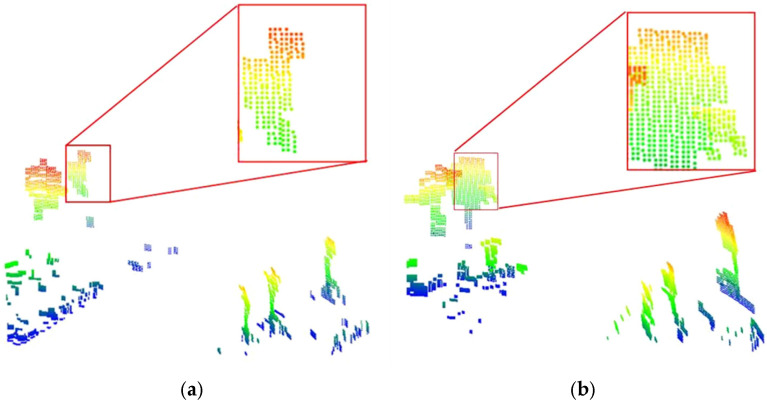
Comparison of point cloud completion effects before and after in Sparse-SemanticKITTI dataset, (**a**) pre-completion, (**b**) post-completion.

In addition, we also provide a qualitative analysis of the point cloud completion results. Figure 6 illustrates the point cloud representations before and after processing with the proposed straight flow completion method. Here, specific target regions are locally magnified to highlight the completion effects. The results in Figure 6 demonstrate significant and substantial completion performance, which can provide adequate data foundation for subsequent point cloud semantic segmentation, further verifying the necessity of the straight flow completion processing.

**Figure 7:**
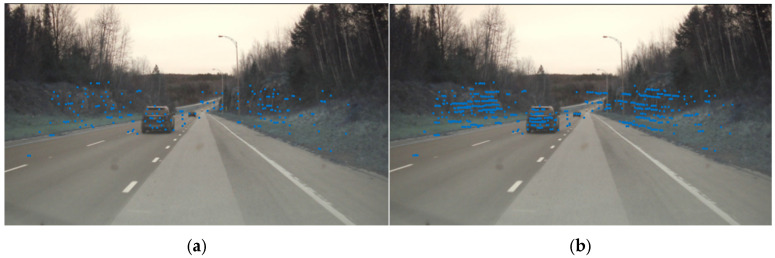
Comparison of point cloud completion effects before and after in RADIal dataset, (**a**) pre-completion, (**b**) post-completion.

Figure 7 displays the imaging results of the measured radar point cloud data from the RADIal dataset for the same scene, both before and after the completion processing. It can be clearly observed that after point cloud completion, the point cloud resolution is enhanced, with particularly noticeable improvement in the point cloud imaging of vehicle target locations. Simultaneously, due to the abundance of grass and trees on both sides of the road, the number of points in the roadside areas also increases significantly. In the original data before point cloud completion, the point cloud is too sparse to effectively support subsequent tasks, such as object detection, further demonstrating the necessity of the point cloud completion module designed in the proposed method.

### 4.3. The Effect of Semantic Segmentation

To evaluate the performance of the proposed method in semantic segmentation, this section conducts comparative experiments on the segmentation effects for both static and dynamic scenarios within the SemanticKITTI dataset. The comparative methods selected include various classical point cloud semantic segmentation models. These comprise models based on single-frame point clouds, such as KPConv [13] and Cylinder3D [8], as well as specifically designed methods for multi-frame point cloud semantic segmentation, including SpSequenceNet [38], TemporalLidarSeg [39], TemporalLatticeNet [44], MarS3D [45], and SegNet4D [43]. The static scenario comparison involves a diverse set of target categories, while the experiment in dynamic scenarios primarily focuses on the semantic segmentation effectiveness for moving objects. The evaluation metric used for semantic segmentation is the mIoU. The comparative results are presented in Table 4 and Table 5, respectively. Analysis of these results demonstrates the adaptability of the proposed method to different scenario targets. Furthermore, the results in Table 5 reflect the capability of the proposed method in extracting motion features.

First, the comparative results in Table 4 show that MemorySEG, SVQNet, 2DPASS, and the proposed method each achieve optimal semantic segmentation performance for specific object categories. Notably, for segmenting the “Truck” category, the proposed method demonstrates a significant advantage, outperforming the second-best method, MemorySEG, by at least 10.9%. On average, 2DPASS shows a clear overall advantage, while the proposed method closely follows. Therefore, in static scenes, the semantic segmentation performance of the proposed method surpasses most classical methods and remains competitive with the top-performing approach.

As seen in Table 5, the proposed method achieves favorable semantic segmentation results for various moving objects, particularly excelling in the “Car” category, where it attains the highest mIoU among all methods. Furthermore, its average segmentation performance across multiple categories is the best overall. Evidently, the incorporation of the motion feature extraction module effectively enhances the representation of motion features, granting the proposed method a distinct advantage in segmenting moving objects.

**Figure 8:**
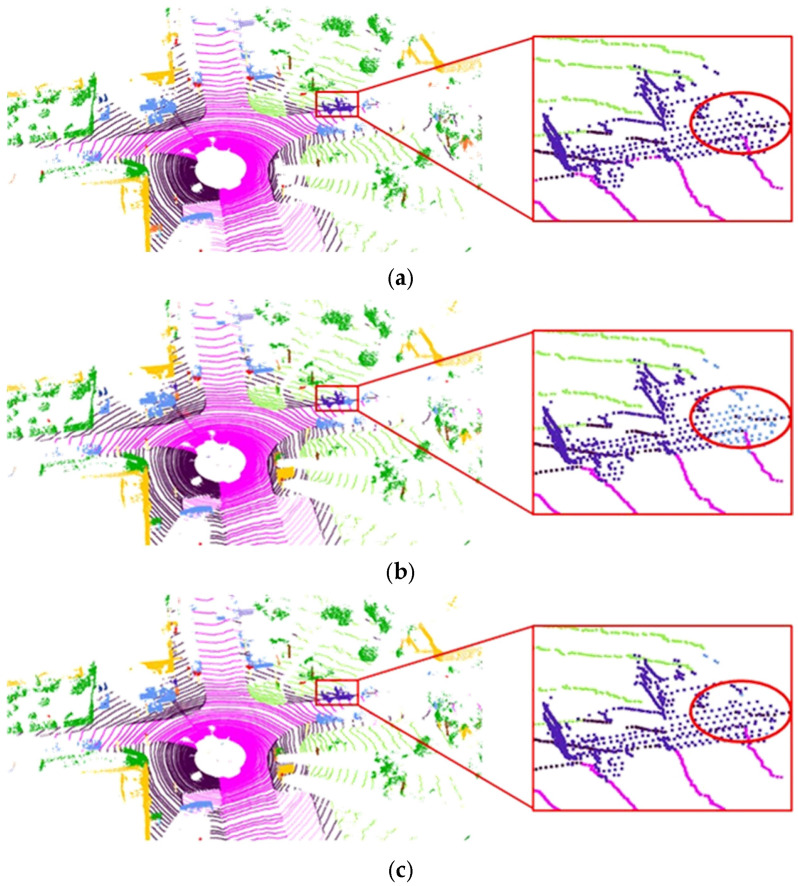
Comparison of point cloud semantic segmentation results with or without multi-branch feature extraction, (**a**) ground truth, (**b**) without instance feature extraction module, (**c**) with instance feature extraction module (proposed).

An additional innovation of our method is the design of multi-branch fusion processing, which effectively integrates instance features with semantic features, thereby enhancing feature representation and improving overall semantic segmentation performance. Figure 8 illustrates the semantic segmentation results both with and without the proposed multi-branch fusion processing on the same dataset, featuring a magnified local region for clearer comparison. The results reveal that, particularly in the area highlighted by the red circle, the semantic segmentation outcomes of our method, after the incorporation of multi-branch fusion processing, align more closely with the ground truth compared to the results obtained without this processing. This finding further underscores the necessity of multi-branch fusion processing.

An ablation study is conducted on the proposed model to specifically evaluate the contributions of the motion feature extraction, instance feature extraction, and adaptive fusion modules. This ablation experiment compared the final semantic segmentation performance in both single-target and multi-target scenarios, with the results presented in Table 6. The baseline model, which does not incorporate the motion feature extraction, instance feature extraction, or adaptive fusion modules, involved processing the sparse point cloud completion results directly through the semantic feature branch of the proposed method to obtain the semantic segmentation result. The results in Table 6 indicate that the inclusion of either the motion feature extraction module or the instance feature extraction module leads to an improvement in semantic segmentation performance of approximately 10% in both the single-target and multi-target scenarios, highlighting the significant role of these modules. Moreover, the simultaneous incorporation of motion feature extraction, instance feature extraction, and adaptive fusion modules yields optimal semantic segmentation performance, demonstrating an improvement of nearly 15% over the use of point cloud completion processing alone. Additionally, comparing the single-target and multi-target scenarios reveals that, regardless of the combination of the key feature extraction modules, the semantic segmentation performance is consistently superior in the single-target scenario. However, when all modules are integrated, the performance improvement in the multi-target scenario is more substantial, demonstrating that the proposed method is particularly effective for semantic segmentation in multi-object scenarios.

**Figure 9:**
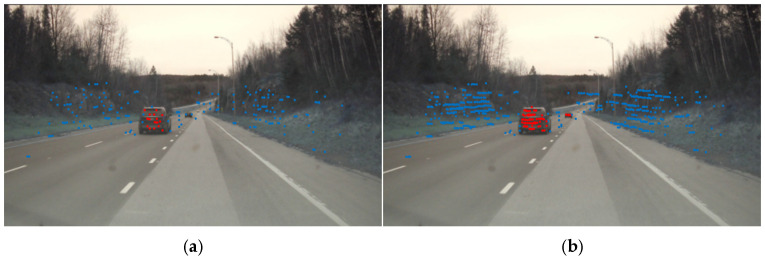
Comparison of semantic segmentation performance before and after point cloud completion on the RADIal dataset, (**a**) pre-completion, (**b**) post-completion.

Finally, Figure 9 presents the semantic segmentation results for a set of data from the RADIal dataset, comparing the outcomes before and after point cloud completion. It can be observed that both achieve relatively accurate semantic segmentation results. However, with the inclusion of the completion process, the point cloud becomes denser, intuitively leading to superior segmentation performance. Particularly for identifying smaller vehicle targets ahead, the pre-completion point cloud in that area is too sparse for accurate segmentation of the vehicle. After completion processing, multiple points are generated in this region, enabling the vehicle area to be correctly identified post-segmentation. This enhances the semantic segmentation accuracy for vehicle targets and further demonstrates the necessity of the point cloud completion module.

In Table 7, we provide a quantitative analysis of the network complexity. The complexity of our model is comparable to that of SegNet4D [44]. However, owing to its superior performance in semantic segmentation, the advantages of our model are more pronounced.

## 5. Conclusions

To address the challenge of poor semantic segmentation performance for sparse point clouds in motion scenarios, this paper proposes a semantic segmentation method based on straight flow point cloud completion and multi-feature fusion. The method connects the point cloud completion and semantic segmentation modules in an end-to-end manner, enhancing its adaptability to point clouds of varying resolutions. During the point cloud completion stage, a straight flow learning strategy is employed to accelerate model parameter training. In the semantic segmentation stage, motion feature extraction and the fusion of instance and semantic features are utilized to improve the model’s capability in capturing and processing information of moving targets. Experimental validation is conducted using point cloud data from the SemanticKITTI and RADIal datasets. For analyzing point cloud completion performance, the LiDAR point clouds in the SemanticKITTI dataset are downsampled to generate sparse inputs, with the original point clouds serving as completion labels. The proposed method is qualitatively and quantitatively demonstrated to outperform classical approaches, showing an improvement of 8.62 in Precision compared to the best classical method. The millimeter-wave radar point clouds in the RADIal dataset are inherently sparser than LiDAR data. After processing with the proposed completion method, the point cloud density is significantly enhanced. In terms of semantic segmentation analysis, the method is evaluated separately on static and dynamic scenes. Although the average segmentation performance in static scenes is not the absolute best, it remains competitive with the top-performing method, i.e., 2DPASS. However, in dynamic scenario, the proposed method achieves the best average performance across multiple object categories, benefiting from motion feature extraction and multi-scale feature fusion. Furthermore, ablation studies are conducted to analyze the necessity of the motion feature extraction, instance feature extraction, and adaptive fusion modules. The experimental results confirm that each module is indispensable. Notably, incorporating either the motion feature extraction module or the instance feature extraction module improves semantic segmentation performance by approximately 10% in both single-target and multi-target scenarios, underscoring the importance of these components. In summary, the proposed semantic segmentation method for sparse point clouds not only adapts effectively to low-resolution point clouds but also achieves more accurate semantic segmentation for targets in dynamic scenes, providing a technical foundation for information perception in complex scenarios such as autonomous driving. In the future, we intend to utilize low-channel LiDAR to gather data in autonomous driving scenarios and perform experiments to evaluate the semantic segmentation performance on real sparse point clouds within dynamic environments.

## Figures and Tables

**Table 1:** Experimental parameter configuration.

Parameters	Value
Batch size	5
Learning rate	0.01
Epoch	120

**Table 2:** Experimental environment.

Items	Parameters
CPU	Intel i7-6700K
GPU	NVIDIA GTX2080Ti 11GB
System environment	Ubuntu 18.0.4
Deep learning framework	Pytorch 1.4.0 cuda10.0 + KNN_CUDA 0.2.0

**Table 3:** Quantitative comparison of Sparse-SemanticKITTI point cloud data.

Metrics	SoftFlow [31]	PointFlow [32]	PVD [30] (*N* = 1000)	Proposed
CD	11.01	10.33	15.42	10.17
HD	712.81	918.57	711.03	655.04
EMD	1.198	1.180	1.030	1.010
FPD	9.91	6.41	6.84	1.87
Precision	42.93	53.22	64.43	73.05
Recall	53.3	53.27	38.09	53.71
F1-score	47.56	53.24	59.78	64.39

**Table 4:** A comparison of semantic segmentation results in static scenes in the SemanticKITTI dataset (mIoU/%).

Method	TemporalLidarSeg [38]	TemporaiLatticNet [44]	KPConv [13]	Meta-RangeSeg [46]	Cylinder3D [8]	Cluster3DSeg [47]	MemorySEG [48]	SVQNet [40]	2DPASS [41]	MarS3D [45]	WaffleIron [49]	SegNet4D [44]	Proposed
Type													
Car	92.1	91.6	93.7	90.8	94.6	95.3	94.0	96.1	96.2	95.1	96.0	94.6	96.7
Bicycle	47.7	35.4	44.9	50.0	67.6	55.9	68.3	64.4	63.6	49.2	69.0	49.4	59.3
Motorcycle	40.9	36.1	47.2	49.5	63.8	52.9	68.8	60.3	63.7	49.5	66.8	49.3	66.4
Truck	39.2	26.9	42.5	29.5	41.3	42.7	51.3	40.4	48.2	39.7	39.9	35.7	62.2
Other-vehicle	35.0	23.0	38.6	34.8	38.8	38.7	40.9	60.9	52.7	36.6	42.3	42.1	59.3
Person	14.4	9.4	21.6	16.6	12.5	15.5	27.0	27.4	35.4	16.2	33.4	9.3	33.8
Bicyclist	0.0	0.0	0.0	0.0	1.7	0.0	0.3	0.0	7.9	1.2	0.4	0.0	6.9
Motorcyclist	0.0	0.0	0.0	0.0	0.2	3.0	2.8	0.0	62.0	0.0	0.0	14.0	15.0
Road	91.8	91.5	86.5	90.8	90.7	91.4	89.9	93.2	89.7	89.9	90.6	92.1	91.4
Sidewalk	75.8	75.3	70.5	74.8	74.5	76.9	74.8	80.5	74.7	74.3	75.3	76.7	75.6
Building	89.8	89.6	90.8	89.8	92.6	91.4	92.2	93.7	93.6	92.1	93.3	92.2	93.1
Terrain	63.8	65.3	64.5	62.1	66.0	66.1	69.3	72.6	72.9	68.2	70.6	69.1	71.5
Vegetation	82.3	84.6	84.6	82.8	85.8	86.5	84.8	87.3	86.2	86.0	86.6	84.9	87.0
Trunk	62.5	66.7	70.3	65.7	72.0	72.7	75.1	76.7	73.9	72.1	73.6	70.0	74.3
Pole	52.6	57.2	57.0	56.2	63.1	64.0	65.5	68.4	65.0	62.8	63.9	59.1	66.3
Traffic-sign	60.4	60.4	53.9	64.5	61.4	68.0	68.5	71.0	70.5	64.8	69.0	63.6	71.3
Average	53.0	50.8	54.1	53.6	57.9	57.6	60.8	62.1	66.1	55.7	60.5	56.2	64.3

**Table 5:** A comparison of semantic segmentation results in motion scenes in the SemanticKITTI dataset (mIoU/%).

Type	Car	Truck	Other-Vehicle	Person	Bicyclist	Motorcycle	Average
Methods							
TemporalLidarSeg [39]	68.2	42.8	40.4	12.9	12.4	2.1	29.8
TemporaiLatticNet [44]	59.7	41.7	51.0	48.8	5.9	0.0	34.5
KPConv [13]	69.4	67.4	67.5	47.2	4.7	5.8	43.7
Meta-RangeSeg [46]	69.0	60.4	57.9	22.0	16.6	2.6	38.1
Cylinder3D [8]	74.9	68.3	65.7	11.9	0.1	0.0	36.8
Cluster3DSeg [47]	81.7	68.2	61.8	46.0	11.2	42.7	51.9
MemorySEG [48]	71.7	74.4	71.7	73.9	15.1	13.6	53.4
SVQNet [40]	80.5	72.4	84.7	91.0	7.5	3.9	56.5
2DPASS [41]	82.1	71.2	80.3	73.1	3.8	16.1	54.4
MarS3D [45]	78.4	67.3	58.0	36.3	10.0	5.1	42.5
WaffleIron [49]	84.2	76.5	70.8	45.5	20.8	24.7	53.7
SegNet4D [44]	78.2	71.5	37.6	55.9	4.0	62.4	51.6
Proposed	85.5	72.3	76.6	64.6	35.5	50.9	64.2

**Table 6:** Ablation experiments of the point cloud semantic segmentation module in single-target and multi-target scenarios (mIoU/%).

Point Cloud Completion	Motion Feature Extraction	Instance Feature Extraction	Adaptive Fusion	Single-Target Scenario	Multi-Target Scenario
√				48.59	43.15
√	√			56.34	53.14
√		√		57.12	53.72
√	√	√		61.45	59.88
√	√	√	√	64.12	63.22

**Table 7:** Network complexity analysis.

Methods	Average Time Used by the Forward Pass (ms)	Maximum Memory Used During Training (GB)
KPConv [13]	225	15.2
WaffleIron [49]	198	16.6
SegNet4D [44]	140	18.1
Proposed	155	17.5

## Data Availability

The original contributions presented in this study are included in the article. Further inquiries can be directed to the corresponding author.
